# Pro-inflammatory adjuvant properties of pigment-grade titanium dioxide particles are augmented by a genotype that potentiates interleukin 1β processing

**DOI:** 10.1186/s12989-017-0232-2

**Published:** 2017-12-08

**Authors:** Sebastian Riedle, Laetitia C. Pele, Don E. Otter, Rachel E. Hewitt, Harjinder Singh, Nicole C. Roy, Jonathan J. Powell

**Affiliations:** 1 Food Nutrition & Health Team, Food & Bio-based Products Group, AgResearch, Grasslands Research Centre, Tennent Drive, Private Bag 11008, Palmerston North, 4442 New Zealand; 2 Riddet Institute, Massey University, Private Bag 11222, Palmerston North, 4442 New Zealand; 3 Present address: Conreso GmbH, Neuhauser Str. 47, 80331, München, Germany; 4 Biomineral Research Group, MRC Human Nutrition Research, Elsie Widdowson Laboratory, 120 Fulbourn Road, Cambridge, CB1 9NL UK; 5 Present address: Center for Dairy Research, University of Wisconsin-Madison, 1605 Linden Drive, Madison, WI 53706-1565 USA; 6 Department of Veterinary Medicine, Biomineral Research Group, University of Cambridge, Madingley Road, Cambridge, CB3 0ES UK

**Keywords:** Nano, Particle, TiO_2_, E171, NOD2, IL-1β, TNF-α, Muramyl dipeptide, Peptidoglycan

## Abstract

**Background:**

Pigment-grade titanium dioxide (TiO_2_) particles are an additive to some foods (E171 on ingredients lists), toothpastes, and pharma−/nutraceuticals and are absorbed, to some extent, in the human intestinal tract. TiO_2_ can act as a modest adjuvant in the secretion of the pro-inflammatory cytokine interleukin 1β (IL-1β) when triggered by common intestinal bacterial fragments, such as lipopolysaccharide (LPS) and/or peptidoglycan.

Given the variance in human genotypes, which includes variance in genes related to IL-1β secretion, we investigated whether TiO_2_ particles might, in fact, be more potent pro-inflammatory adjuvants in cells that are genetically susceptible to IL-1β-related inflammation.

**Methods:**

We studied bone marrow-derived macrophages from mice with a mutation in the nucleotide-binding oligomerisation domain-containing 2 gene (*Nod2* ^m/m^), which exhibit heightened secretion of IL-1β in response to the peptidoglycan fragment muramyl dipeptide (MDP). To ensure relevance to human exposure, TiO_2_ was food-grade anatase (119 ± 45 nm mean diameter ± standard deviation). We used a short ‘pulse and chase’ format: pulsing with LPS and chasing with TiO_2_ +/− MDP or peptidoglycan.

**Results:**

IL-1β secretion was not stimulated in LPS-pulsed bone marrow-derived macrophages, or by chasing with MDP, and only very modestly so by chasing with peptidoglycan. In all cases, however, IL-1β secretion was augmented by chasing with TiO_2_ in a dose-dependent fashion (5–100 μg/mL). When co-administered with MDP or peptidoglycan, IL-1β secretion was further enhanced for the *Nod2* ^m/m^ genotype. Tumour necrosis factor α was triggered by LPS priming, and more so for the *Nod2* ^m/m^ genotype. This was enhanced by chasing with TiO_2_, MDP, or peptidoglycan, but there was no additive effect between the bacterial fragments and TiO_2_.

**Conclusion:**

Here, the doses of TiO_2_ that augmented bacterial fragment-induced IL-1β secretion were relatively high. In vivo, however, selected intestinal cells appear to be loaded with TiO_2_, so such high concentrations may be ‘exposure-relevant’ for localised regions of the intestine where both TiO_2_ and bacterial fragment uptake occurs. Moreover, this effect is enhanced in cells from *Nod2* ^m/m^ mice indicating that genotype can dictate inflammatory signalling in response to (nano)particle exposure. In vivo studies are now merited.

**Electronic supplementary material:**

The online version of this article (10.1186/s12989-017-0232-2) contains supplementary material, which is available to authorized users.

## Background

Potential toxicological effects following exposure to titanium dioxide (TiO_2_) are of current interest [1, 2]. TiO_2_ is a mineral pigment which, when used in a particulate form, is valued for its properties as a whitening or brightening agent, and is included in some processed foods (E171 on ingredients lists), toothpastes, capsules and tablets. From these sources, the average daily intake of pigment-grade TiO_2_ for an adult in the UK is about 10^12^ particles/day [3, 4], nominally ~0.04 mg/kg/day for a 70 kg adult. These findings are supported by a recent Dutch study with mean long term intakes of pigment-grade TiO_2_ ranging from 0.06 mg/kg/day in elderly subjects to 0.17 mg/kg/day for 7–69-year-olds [5]. In 2–6 year old children, however, it was higher at 0.67 mg/kg/day [5].

It is well established that particles of TiO_2_, likely derived from sources of the Western lifestyle described above, accumulate in certain cells, such as macrophages in Peyer’s patches of the human small intestine [6–10]. Whether they have any deleterious impact in this environment remains a matter of speculation, but, if they do, both cell accumulation and host factors are likely to be important [4]. Indeed, it has been often noted that the accumulation of these particles occurs where the earliest signs of Crohn’s disease have been reported [11]. With respect to cell accumulation and stimulation, the pristine particle is probably of limited relevance. The intestinal lumen is a ‘soup’ of proteins, bacterial fragments, ions, small organic molecules etc. and these will modify the surface of the particles through adsorptive interactions. Consistent with this, there are several reports of how TiO_2_ particles act as an adjuvant for cellular responses to the bacterial-derived molecule lipopolysaccharide (LPS), either through formation of ‘conjugates’ or by co-incubation [12–15].

More recently it has been shown that pigment-grade TiO_2_ is a modest trigger of the NLR family pyrin domain-containing 3 (NLRP3) inflammasome and that this activity may contribute to intestinal inflammatory properties of the particle in murine models [16]. The inflammasome regulates the activation of caspase-1 which, in turn, determines cleavage of inactive pro-interleukin 1β (pro-IL-1β) to form mature pro-inflammatory interleukin 1β (IL-1β). If such a pro-inflammatory effect from oral TiO_2_ exposure translates significantly from murine models to humans, it must be occurring in a small minority of the population because most children and adults do not have intestinal disease. In this respect some variants of human genotype could be important. Indeed, it is well recognised that inflammatory bowel diseases are complex polygenic disorders [17]. Certain mutations in the nucleotide-binding oligomerisation domain-containing 2 (*NOD2*) gene, for example, are associated with an increased risk of the inflammatory bowel disease, Crohn’s disease [18, 19]. Maeda et al. have shown that in mice at least one form of *Nod2* mutation potentiates IL-1β processing and enhances risk of intestinal inflammation [20]. These mice carry a known Crohn’s disease-associated ‘knockin’ mutation in the *Nod2* locus but also carry a duplication of the 3′ end of the wild-type (WT) *Nod2* locus [21], and herein are designated as *Nod2* ^m/m^ mice. Specifically, development of a modest pro-inflammatory phenotype in these animals is reportedly triggered by a bacterial peptidoglycan moiety, muramyl dipeptide (MDP), in an IL-1β-dependent fashion [20]. Since bacterial peptidoglycan is taken up by Peyer’s patch phagocytes [22, 23] it raises the possibility that TiO_2_ could act as an adjuvant for the pro-inflammatory effects of peptidoglycan, and especially so where the genotype potentiates IL-1β processing. Hence, using bone marrow-derived macrophages (BMDMs) from WT and *Nod2* ^m/m^ mice, we have tested these possibilities using an assay of short ‘pulse and chase’ format, to determine if and how TiO_2_ could amplify IL-1β secretion at the cellular level.

## Methods

### Study design

The macrophage-stimulatory effects of dietary TiO_2_ were investigated, either alone or in combination with microbial-associated molecular patterns (MAMPs), using cells from WT and *Nod2* ^m/m^ mice. MAMP concentrations were fixed whereas a range of TiO_2_ concentrations was investigated. LPS pre-stimulation of cells was employed as this MAMP is abundant in the intestinal lumen and can prime cells for an inflammasome-driven response (IL-1β secretion), as described in the Introduction. Parameters assessed were overall cell viability, particle uptake, and secretion of the pro-inflammatory cytokines IL-1β and tumour necrosis factor alpha (TNF-α).

### TiO_2_ particles

Food- and pharmaceutical-grade TiO_2_ particles with anatase crystal structure and a purity of not less than 99% were obtained from Sensient Colors (St. Louis, USA). According to the manufacturer, the TiO_2_ particles had an average particle size of 300 nm and a maximum particle size of 1.0 μm, which had been determined using a sediograph instrument. We undertook further analysis of the powder, initially with transmission electron microscopy. A 1 mg/mL suspension of TiO_2_ powder in distilled water (Life Technologies, Auckland, New Zealand) with 0.5% bovine serum albumin (BSA; Life Technologies) as a dispersant was prepared. A drop of the TiO_2_ particle suspension was placed on a 200-mesh carbon-coated copper grid, and excessive liquid was absorbed with filter paper. The particles were analysed with a Philips CM10 transmission electron microscope at 80 kV. The image analysis software iTEM (Olympus Soft Imaging Solutions, Münster, Germany) was used to record the images digitally and subsequently measure the diameter of the particles.

In addition, particle size under cell culture conditions was determined with nanoparticle tracking analysis, which is a method to analyse dispersed particles based on their Brownian motion, similar to analysis with dynamic light scattering [24]. A 100 μg/mL TiO_2_ particle suspension was prepared in tissue culture medium (TCM) consisting of RPMI 1640 medium (Sigma-Aldrich, Gillingham, UK) with 10% foetal bovine serum (FBS; PAA Laboratories, Yeovil, UK) and 1% penicillin-streptomycin antibiotics (Sigma-Aldrich). The suspension was sonicated for 10 min to facilitate distribution of the TiO_2_ particles in the medium. The motion of the particles in suspension was digitally recorded with a NanoSight NS500 instrument (NanoSight, Amesbury, UK). Three TiO_2_ suspensions were analysed independently. The particle sizes were calculated from the recorded videos with nanoparticle tracking analysis software (Nanosight).

### Animals

For the cell culture experiments, bone marrow was obtained from 10 to 18 week old female C57BL/6 WT and *Nod2* ^m/m^ mice. The original WT breeding pairs were purchased from the Jackson Laboratory (Bar Harbor, USA) and bred at the AgResearch Small Animal Colony (Hamilton, New Zealand). Breeding pairs for *Nod2* ^m/m^ mice on a C57BL/6 background were kindly provided by Lars Eckmann [20], and backcrossed with WT mice for 10 generations at the AgResearch Small Animal Colony. The mice were kept under conventional conditions at all times [25].

### Harvest of BMDMs and cell culture

For the bone marrow collection, the mice were euthanised with CO_2_ asphyxiation and cervical dislocation. Femurs and tibias were collected, sterilised in 70% ethanol for 10 s, and the bone marrow flushed out with cold RPMI 1640 medium (Life Technologies). Single cell suspensions were prepared by passing the cells repeatedly through a 19G needle (BD Biosciences, Singapore) and a 70 μm cell strainer (BD Labware, Franklin Lakes, USA). Bone marrow cells were re-suspended in TCM consisting of RPMI 1640 medium (Life Technologies) with 10% FBS (Life Technologies), 1% penicillin-streptomycin antibiotics (Life Technologies), and 10 μg/mL macrophage colony-stimulating factor (eBioscience, San Diego, USA). The cells were transferred to non-tissue culture treated 24-well plates (BD Labware) at a concentration of 1 × 10^6^ cells/well in 1 mL TCM and cultured at 37 °C in 7% CO_2_/93% air. Half of the TCM was replaced every 3 days with fresh TCM throughout the culture period. Bone marrow cells were fully differentiated into BMDMs on day 7 and used for experiments between day 8 and day 10.

### Stimulation of BMDMs with TiO_2_ particles +/− peptidoglycan or MDP

As previously noted, a short ‘pulse (LPS) and chase (TiO_2_ +/− peptidoglycan or MDP)’ format was used to dissect out the point in the pathway that the particles might act as pro-inflammatory adjuvants of MAMPs. To that effect, harvested murine BMDMs from each genotype, +/− LPS pre-stimulation, were exposed to a range of TiO_2_ particle concentrations +/− peptidoglycan or MDP, as detailed below.

To activate the cells, especially for pro-IL-1β induction, BMDMs were first primed in culture with 1 mL TCM containing 10 ng/mL LPS from *Escherichia coli* O111:B4 (Sigma-Aldrich, Auckland, New Zealand) for 3 h at 37 °C in 7% CO_2_/93% air. Unprimed BMDMs were cultured under identical conditions but without LPS. All cells were then washed in TCM before the TiO_2_ suspensions were added. A 1 mg/mL TiO_2_ stock suspension was first prepared in distilled water and autoclaved. This stock suspension was used to prepare TiO_2_ suspensions in the TCM with final concentrations from 5 μg/mL to 100 μg/mL. Similar concentrations have been used in previous studies that examined cytokine secretion by phagocytic cells after TiO_2_ exposure [13, 26–28]. The TiO_2_ suspensions were sonicated in a water bath for 10 min before 1 mL of the respective TiO_2_ suspension was added to the cells. When the BMDMs were co-stimulated with MAMPs, either synthetic MDP or peptidoglycan from *Bacillus subtilis* (both from Sigma-Aldrich) was added to the respective TiO_2_ suspensions in TCM, both at a final concentration of 10 μg/mL. The BMDMs were incubated with TiO_2_ particles in TCM with or without the co-stimulants for 3 h at 37 °C in 7% CO_2_/93% air.

### Flow cytometry analysis of BMDMs

Only LPS pre-stimulated BMDMs were used for flow cytometry analysis. After incubation with particle suspensions with or without the other MAMPs, the cells were collected for analysis with flow cytometry. Briefly, cells were washed with TCM, incubated for 30 min with cold phosphate-buffered saline (PBS; Life Technologies) on ice, and collected by vigorous pipetting. The BMDMs were re-suspended in 150 μL PBS containing 5% FBS, 2% ethylenediaminetetraacetic acid (Life Technologies), and 1% sodium azide (BDH Laboratory Supplies, Poole, UK). The cells were first incubated for 15 min on ice with 1 μg/mL anti-mouse CD16/32 blocking antibody (clone 93; BioLegend, San Diego, USA) and then stained for 15 min on ice with 1 μg/mL anti-mouse phycoerythrin-labelled F4/80 antibody (clone BM8; BioLegend), a specific marker for murine macrophages. In addition, 0.8 μg/mL propidium iodide (PI; Life Technologies) was added to each sample immediately before analysis for viability assessment. The cells were analysed with a FACS Calibur flow cytometer (BD Biosciences, San Jose, USA), and at least 12,000 events per sample were acquired with the CellQuest Pro software (BD Biosciences). Data analysis was performed with FlowJo (Tree Star, Ashland, USA). For details on the gating strategy see Additional file 1. The percentage of viable cells in relation to the total number of detected events was assessed with PI staining. Cells that did not show PI staining (PI^−^) were considered to be viable cells. BMDMs were identified among the PI^−^ cells based on the expression of F4/80, i.e. viable cells that expressed F4/80 (PI^−^F4/80^+^) were classified as viable BMDMs. The percentages of PI^−^F4/80^+^ cells in relation to the total number of viable cells are shown in Additional file 2. Uptake of TiO_2_ particles by BMDMs was assessed with the median side scatter (SSC) intensity of the PI^−^F4/80^+^ cell populations. According to previous studies, an increase in SSC intensity indicated TiO_2_ particle uptake [12, 29, 30].

### Validation of SSC analysis by flow cytometry as a measure of TiO_2_ cellular uptake

To confirm that increases in SSC intensity did indeed indicate TiO_2_ particle uptake, we undertook correlative studies with conventional flow cytometry and imaging cytometry which allows visualisation of TiO_2_ uptake by individual cells [31]. This technique was not available in the laboratory that undertook the above work and is impractical for a very large number of samples, so only the lower concentration range was investigated and correlated to ensure true discrimination from background.

To quantify TiO_2_ cellular uptake (i.e. association and localisation) by peripheral myeloid cell populations, fresh leukocyte cones were purchased from the National Blood Service (Cambridge, UK). Peripheral blood mononuclear cells (PBMCs) were isolated by density centrifugation using the separating medium Lymphoprep (Axis Shield Diagnostics, Dundee, UK) and frozen until use. PBMCs from 3 leucocyte cones were thawed and rested for 2 h prior to incubation at 1 × 10^6^ cells/mL with 0 μg/mL, 5 μg/mL, or 10 μg/mL TiO_2_ and incubated for 24 h in RPMI 1640 medium (Sigma-Aldrich, Gillingham, UK) supplemented with 10% FBS (Sigma-Aldrich).

After incubation, cells were washed with ice cold PBS (Sigma-Aldrich) containing 1% BSA (Sigma-Aldrich) and stained for the human monocyte/myeloid cell markers CD14 Alexa Fluor 488 or CD11c fluorescein isothiocyante (both from BD Biosciences), respectively. Single stain compensation tubes and unstained PBMC tubes, with and without TiO_2_, were also prepared at this time from PBMC samples for the generation of compensation matrices. After staining, PBMCs were washed with ice cold PBS containing 1% BSA, re-suspended in a small volume of PBS containing 2% paraformaldehyde (Sigma-Aldrich) solution, and placed on ice in the dark until acquisition.

Imaging cytometry analysis was undertaken using an ImageStreamX Mark I platform (Amnis-Merck-Millipore, Seattle, USA), equipped with 405 nm and 488 nm lasers for excitation, a 785 nm laser for a scatter signal with standard filter sets, multi magnification (20×/40×/60×) and extended depth of field. INSPIRE software (Amnis) was used for acquisition and IDEAS software (Amnis) for analysis. The machine passed all tests and was fully calibrated prior to acquisition of samples. Before acquisition, cells were filtered through 35 μm cell strainers (BD Labware). A minimum of 10,000 events per sample were acquired. Compensation matrices were generated by running single stained cells (i.e. single cell surface marker) and analysed using IDEAS software. For analysis, TiO_2_ positive cells were identified and quantified using a spot count analysis of dark spots appearing within the cells based on bright-field images of CD14 positive (CD14^+^) cells. Briefly, cells were first plotted as area versus aspect ratio of the bright-field images and a single cell gate drawn, followed by a focused gate. CD14^+^ cells were then gated based on fluorescence intensity. A custom dark spot count mask was generated to quantify CD14^+^ cells, with cells positive for 2 or more darks pots gated as dark spot positive.

Conventional flow cytometry analysis was performed using a CyAn ADP 9 colour analyser (Beckman Coulter, Brea, USA) equipped with 405 nm, 488 nm and 642 nm solid-state lasers and 11 detectors in standard configuration. Summit software was used for acquisition and analysis (Beckman Coulter, USA). At least 500,000 events were acquired on the flow cytometer using a lowered SSC setting on a logarithmic scale. Samples were filtered through 35 μm cell strainers (BD Labware) directly prior to acquisition. For data analysis, events were first plotted as forward versus side scatter using SSC on a logarithmic scale, and a large gate was drawn excluding debris. Cells were then further gated for CD11c positivity based on fluorescence intensity for the mean fluorescence intensity (MFI) of the SSC signal of CD11c^+^ myeloid cells.

### Stimulation of PBMCs with monosodium urate crystals or silica nanoparticles

We confirmed that other exemplar inflammasome-activating particles to which humans are exposed, namely monosodium urate (MSU) crystals and silica nanoparticles (SNPs) [32, 33], promote IL-1β processing in our short ‘pulse and chase’ format. Isolated PBMCs (*n* = 4) were thawed and rested overnight. Cells (1.10^6^ cells/mL) were then subjected to LPS pre-stimulation (10 ng/mL, *Escherichia coli* O111:B4; Sigma-Aldrich) to induce the production of pro-IL-1β or with TCM as a negative control. Following 3 h, cells were washed and then challenged with 100 μg/mL MSU crystals (Caltag Medsystems, Buckingham, UK) or 100 μg/mL SNPs (InvivoGen, San Diego, USA) for a further 3 h. Following this, cells were washed and replenished with fresh TCM for a further 21 h (3 + 21 h). Supernatants were collected at the 3 h and 3 + 21 h time points for IL-1β analyses.

### Cytokine detection in cell supernatants

Cell supernatants were collected at the time points indicated and stored at −20 °C until required for cytokine analysis. IL-1β (TiO_2_ and exemplar inflammasome-activating particles) and TNF-α (TiO_2_ only) were investigated with enzyme-linked immunosorbent assay (ELISA) using DuoSet ELISA kits (R&D Systems, Minneapolis, USA) according to the manufacturer’s instructions. The cytokine concentrations were generally determined with a FlexStation 3 microplate scanner (Molecular Devices, Sunnyvale, USA) and Soft Max Pro software (Molecular Devices).

### Statistical analysis

All statistical comparisons were carried out using R (R Development Core Team, Vienna, Austria). For analysis of the flow cytometry results, the groups according to genotype (WT or *Nod2* ^m/m^ BMDMs) were compared with two-way analysis of variance (ANOVA) using co-stimulation condition and TiO_2_ exposure as the two factors. For analysis of the cytokine secretion results without co-stimulation, the groups according to genotype (WT or *Nod2* ^m/m^ BMDMs) were compared with one-way ANOVA using TiO_2_ exposure as the single factor. For analysis of the cytokine secretion results with co-stimulation, the groups according to co-stimulation condition (MDP or peptidoglycan) were compared with two-way ANOVA using genotype and TiO_2_ exposure as the two factors. In instances where two-way ANOVA results showed a significant interaction effect or the one-way ANOVA results indicated a significant difference between groups, pairwise group comparisons were performed with Tukey’s post-hoc test. Figures depict group means ± standard deviation (SD). Finally, paired T tests were used to compare supernatant levels of IL-1β for cells exposed to MSU crystals or SNPs versus non-particle-exposed control cells. Group means ± standard error of the mean (SEM) are depicted in the corresponding figure.

## Results

### TiO_2_ particle characterisation

**Fig. 1:**
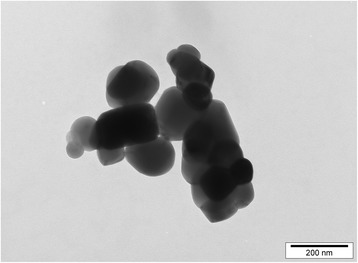
Transmission electron microscopy image of TiO_2_ particles. Food- and pharmaceutical-grade anatase TiO_2_ particles were suspended in distilled water with 0.5% BSA at a concentration of 1 mg/mL. The particle suspension was analysed with transmission electron microscopy at 80 kV. A representative image is shown; scale bar = 200 nm

**Fig. 2:**
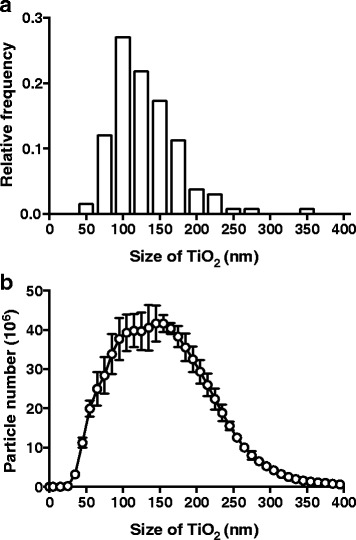
Size determination of TiO_2_ particles. **a** Food- and pharmaceutical-grade anatase TiO_2_ particles were suspended in distilled water with 0.5% BSA at a concentration of 1 mg/mL. The particle suspension was analysed with transmission electron microscopy at 80 kV. TiO_2_ particle diameters were measured with image analysis software. The distribution of the particle diameters, grouped in sizes of 25 nm, is shown as a relative frequency histogram, *n* = 133. **b** Food- and pharmaceutical-grade anatase TiO_2_ particles were suspended in RPMI 1640 medium with 10% FBS and 1% penicillin-streptomycin at a concentration of 1 mg/mL. TiO_2_ particle sizes were determined with nanoparticle tracking analysis, and the size distribution of the particles is plotted as a line graph. Data represent mean ± SD from three independent experiments

Several images of TiO_2_ particles were obtained with transmission electron microscopy and a representative image is shown in Fig. 1. The diameters of individual particles were measured with image analysis software. The average primary particle size was 119 nm with a SD of 45 nm, and the observed particle sizes ranged from 50 nm to 350 nm with a maximum frequency at 100 nm (Fig. 2a). Approximately 54% of the particles had a diameter between 125 nm and 200 nm, and about 40% had a diameter of 100 nm or less.

TiO_2_ particles were suspended in TCM for the subsequent cell culture experiments, so the particle sizes in TCM were also investigated, using nanoparticle tracking analysis. According to this method, the average particle size was 160 nm, and the sizes ranged between 20 nm and 450 nm (Fig. 2b). Approximately 20% of the particles had a diameter of less than 100 nm. The slight increase in particle sizes versus electron microscopy measures probably results from the differing environments as, in solution, particles have a hydration shell and are liable to adsorb TCM molecules. However, the possibility of a small degree of agglomeration in this environment cannot be precluded.

### Cellular effects of TiO_2_ particles

As intended with our short ‘pulse and chase’ style assay, BMDMs of both genotypes that were not primed with LPS (i.e. sham-pulsed) did not secrete meaningful amounts of IL-1β when chased for 3 h with TiO_2_ from 0 μg/mL to 100 μg/mL +/− MDP or peptidoglycan (IL-1β secretion always <5 pg/mL; data not shown). All subsequent data therefore refer to results with LPS-primed cells.

#### Cell viability

**Fig. 3:**
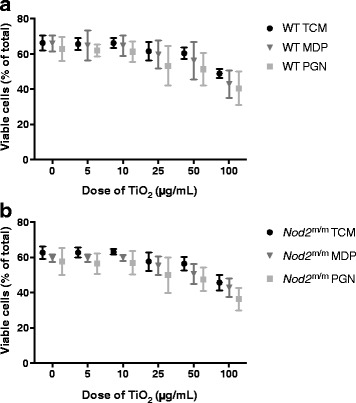
Viability of LPS-primed BMDMs after chasing with TiO_2_ +/− peptidoglycan or MDP. BMDMs from WT (**a**) and *Nod2* ^m/m^ (**b**) mice were pre-stimulated for 3 h with LPS (10 ng/mL). Then BMDMs were incubated for 3 h with the indicated concentrations of TiO_2_ particles suspended in TCM alone (TCM), or TCM + 10 μg/mL MDP (MDP), or TCM + 10 μg/mL peptidoglycan (PGN). Cells were stained with PI and F4/80 antibody for murine macrophages and analysed with flow cytometry, and viability was determined with PI exclusion. Percentages of PI^−^ cells in relation to the total number of detected events are shown. Data represent mean ± SD from two independent experiments with three replicates each, *n* = 6

The viability of LPS-pulsed cells, from WT (Fig. 3a) and *Nod2* ^m/m^ mice (Fig. 3b), was significantly reduced by chasing with TiO_2_ particles, in a dose-responsive fashion (*p* < 0.001 for trend, Fig. 3a and b). Addition of peptidoglycan or MDP during the chase phase marginally, but significantly, decreased cell viability further (*p* < 0.001 for trend), although there was no interaction effect with TiO_2_ exposure (Fig. 3a and b).

#### Particle uptake

**Fig. 4:**
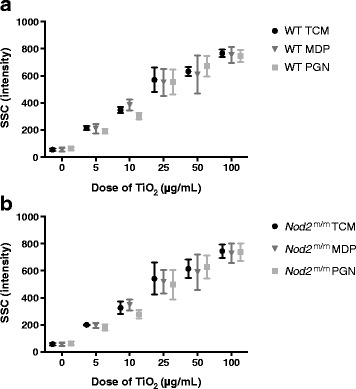
Particle uptake by LPS-primed BMDMs after chasing with TiO_2_ +/− peptidoglycan or MDP. BMDMs from WT (**a**) and *Nod2* ^m/m^ (**b**) mice were pre-stimulated for 3 h with LPS (10 ng/mL). Then BMDMs were incubated for 3 h with the indicated concentrations of TiO_2_ particles suspended in TCM alone (TCM), TCM + 10 μg/mL MDP (MDP), or TCM + 10 μg/mL peptidoglycan (PGN). Cells were stained with PI and F4/80 antibody for murine macrophages and analysed with flow cytometry, and median SSC intensities of PI^−^F4/80^+^ cells were recorded. Data represent mean ± SD from two independent experiments with three replicates each, *n* = 6

**Fig. 5:**
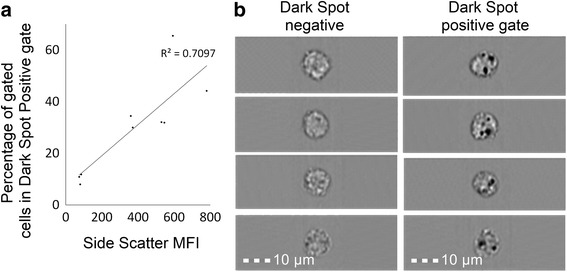
Correlation of SSC intensity and dark spots in bright-field images by flow and imaging cytometry. PBMCs from human blood were incubated for 24 h with 0 μg/mL, 5 μg/mL, or 10 μg/mL TiO_2_ particles in TCM and stained with either CD11c or CD14 antibodies for human monocytes/myeloid cells and analysed with conventional flow or imaging cytometry, respectively. **a** Correlation between increases in SSC MFI of CD11c^+^ myeloid cells identified using conventional flow cytometry and the percentages of CD14^+^ cells bearing dark spots in bright-field measured by imaging cytometry; Pearson correlation *p* < 0.01, *r* = 0.8424. **b** Representative images of cells designated ‘dark spot negative’ or ‘dark spot positive’ by imaging cytometry; scale bar = 10 μm

Particle uptake was assessed by flow cytometric SSC intensities for LPS-pulsed viable (PI^−^) F4/80^+^ WT (Fig. 4a) and *Nod2* ^m/m^ BMDMs (Fig. 4b). During the chase phase, SSC intensities of WT and *Nod2* ^m/m^ BMDMs increased with increasing TiO_2_ concentrations (*p* < 0.001 for trend) but were unaffected by the presence of peptidoglycan or MDP (Fig. 4a and b). To confirm that such increases in SSC intensities did result from TiO_2_ uptake, as anticipated and as previously reported [12, 29, 30], we compared this form of analysis with imaging cytometry which allows visualisation of particle uptake [31]. Using PBMCs, and the lower end of the exposure range (where error would be greatest), increases in SSC intensity of myeloid-gated cells correlated positively and closely with observed TiO_2_ uptake (*r* = 0.84, *p* < 0.01; Fig. 5).

#### IL-1β secretion

**Fig. 6:**
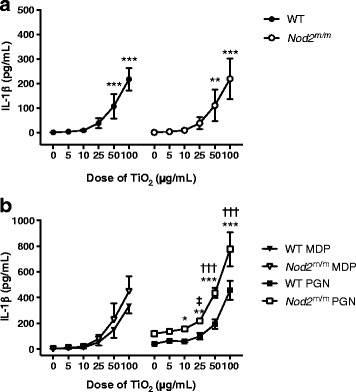
IL-1β secretion by LPS-primed BMDMs after chasing with TiO_2_ +/− peptidoglycan or MDP. BMDMs from WT and *Nod2* ^m/m^ mice were pre-stimulated for 3 h with LPS (10 ng/mL). Then BMDMs were incubated for 3 h with the indicated concentrations of TiO_2_ particles suspended in TCM alone (**a**) or suspended in TCM + 10 μg/mL MDP (MDP) or TCM + 10 μg/mL peptidoglycan (PGN) (**b**). Supernatant concentrations of IL-1β were analysed by ELISA. Data represent mean ± SD from two independent experiments with three replicates each, *n* = 6. **a** Results were analysed with one-way ANOVA and Tukey’s post-hoc test; **p < 0.01, ***p < 0.001 compared to respective WT or *Nod2* ^m/m^ cells incubated without TiO_2_. **b** Results were analysed with two-way ANOVA and Tukey’s post-hoc test; *p < 0.05, **p < 0.01, ***p < 0.001 for *Nod2* ^m/m^ cells compared to WT cells cultured with the same TiO_2_ concentration, ^†††^ *p* < 0.001 for WT and *Nod2* ^m/m^ cells compared to respective WT or *Nod2* ^m/m^ cells incubated without TiO_2_, ^‡^ *p* < 0.05 for *Nod2* ^m/m^ cells compared to *Nod2* ^m/m^ cells incubated without TiO_2_

In LPS-pulsed BMDMs chased with TCM alone (i.e. zero dose TiO_2_ in Fig. 6a), there was no secretion of mature IL-1β, consistent with the role of LPS in stimulating pro-IL-1β but not triggering the inflammasome [34, 35]. Again as anticipated, chasing LPS-primed BMDMs with TiO_2_ led to mature IL-1β secretion in a dose-dependent fashion (*p* < 0.001; Fig. 6a) as these particles are a modest activator of the inflammasome [16, 28]. Pairwise group comparison with Tukey’s post-hoc test indicated significant IL-1β stimulation with TiO_2_ doses in TCM of ≥50 μg/mL (*p* between <0.01 and <0.001; Fig. 6a).

Similarly, chasing LPS-primed BMDMs with TiO_2_ + peptidoglycan or MDP increased IL-1β secretion in a dose-dependent fashion, for cells of both genotypes (*p* < 0.001, Fig. 6b). However, genotype significantly influenced the extent of the IL-1β response (*p* < 0.01 for + MDP and *p* < 0.001 for + peptidoglycan). Furthermore, an interaction effect between genotype and TiO_2_ exposure was observed for peptidoglycan (*p* < 0.001), but not for MDP. Pairwise comparisons between groups with Tukey’s post-hoc test, when chasing with TiO_2_ + peptidoglycan, showed that the amount of IL-1β released by WT and *Nod2* ^m/m^ BMDMs differed significantly when the cells were similarly exposed to ≥10 μg/mL TiO_2_ (*p* between <0.05 and <0.001; Fig. 6b).

#### TNF-α secretion

**Fig. 7:**
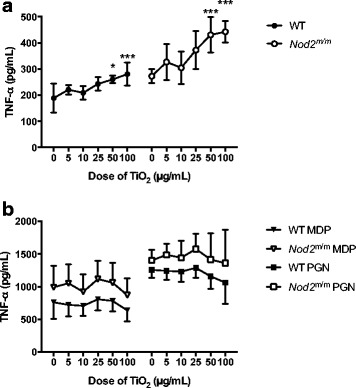
TNF-α secretion by LPS-primed BMDMs after chasing with TiO_2_ +/− peptidoglycan or MDP. BMDMs from WT and *Nod2* ^m/m^ mice were pre-stimulated for 3 h with LPS (10 ng/mL). Then BMDMs were incubated for 3 h with the indicated concentrations of TiO_2_ particles suspended in TCM alone (**a**) or suspended in TCM + 10 μg/mL MDP (MDP) or TCM + 10 μg/mL peptidoglycan (PGN) (**b**). Supernatant concentrations of TNF-α were analysed by ELISA. Data represent mean ± SD from two independent experiments with three replicates each, *n* = 6. **a** Results were analysed with one-way ANOVA and Tukey’s post-hoc test; *p < 0.05, ***p < 0.001 compared to respective WT or *Nod2* ^m/m^ cells incubated without TiO_2_

LPS priming led to marked secretion of TNF-α even when chased with TCM alone (Fig. 7a) because, unlike IL-1β [36], there is no requirement for a second signal to enable protein formation and secretion of this cytokine. Chasing LPS-primed BMDMs with TiO_2_ led to further TNF-α secretion in a dose-dependent fashion (*p* < 0.001; Fig. 7a) and, again, Tukey’s post-hoc test indicated significant TNF-α stimulation with TiO_2_ doses in TCM of ≥50 μg/mL (*p* between <0.05 and <0.001; Fig. 7a).

In contrast to IL-1β, the secretion of TNF-α by LPS-primed BMDMs that were chased with MDP or peptidoglycan was not affected by additional TiO_2_ exposure regardless of dose (i.e. the MAMPs rather than the particles dominated the scene for TNF-α secretion; Fig. 7b).

Although in all cases the genotype had a significant influence (*p* < 0.001) on TNF-α secretion, being greater for cells from *Nod2* ^m/m^ than WT mice, there was no interaction effect between genotype and TiO_2_ exposure (Fig. 7a and b).

#### Specificity of TiO_2_ effect

**Fig. 8:**
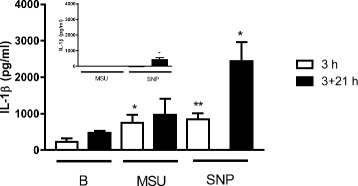
IL-1β secretion by PBMCs following exposure to MSU crystals or SNPs. Secretion of IL-1β by PBMCs with (main figure) or without (inset) initial exposure to 10 ng/mL LPS for 3 h followed by exposure to MSU crystals (100 μg/mL) or SNPs (100 μg/mL) for a further 3 h. The supernatant was either collected immediately for analysis (3 h; open bars) or following a further 21 h of cell incubation in fresh (i.e. without added particles or MAMPs) TCM (3 + 21 h; black bars). All data are expressed as mean ± SEM (*n* = 4). Results were analysed by paired T test in comparison to baseline (B), i.e. non-particle-exposed cells; *p < 0.05 and **p < 0.01 versus respective baseline

Activation of the inflammasome is by no means specific to TiO_2_ particles although Pele et al. have shown that correct design of in vitro experiments is critical. Notably, cell gorging of particles through extended particle exposure (e.g. over 24 h) can lead to false positives [35]. SNPs and MSU crystals are considered exemplar particulate stimulants of the inflammasome, and we confirmed that, with similar short exposures as for our TiO_2_ particles (3 h) and LPS priming, IL-1β secretion was enhanced compared to non-particle-exposed cells (Fig. 8).

## Discussion

### Relevance and context of our findings

The distal intestinal tract is bathed in high concentrations of MAMPs such as LPS and peptidoglycan (and their fragments) due to the continuous turnover of the microbiome. Since ingested particles, such as pigment-grade TiO_2_, are taken up by intestinal cells from this distal environment it is important to consider interactions of these components (i.e. MAMPs + particles) when looking at potential cellular effects. In this work we have further considered the impact of genotype, namely one that imparts greater potential for an inflammatory phenotype (*Nod2* ^m/m^) than the WT version. We confirm that (a) primed cells from *Nod2* ^m/m^ mice secrete higher concentrations of pro-inflammatory cytokines, namely IL-1β and TNF-α, in response to MDP-containing MAMPs than cells from WT mice [20] and (b) TiO_2_ particles are mediators of inflammasome activation [12, 16, 28, 33]. Additionally, we show for the first time that, in primed cells exposed to peptidoglycan, the concentration of TiO_2_ that is required to trigger the inflammasome and induce IL-1β secretion is lower for cells from *Nod2* ^m/m^ mice than it is from WT mice. This may have important implications as discussed below.

It is established that at least some ingested TiO_2_ particles are taken up by intestinal cells, especially by macrophages of large lymphoid follicles of the ileum termed Peyer’s patches [6–10]. Recent data suggest that cells of the large bowel can also scavenge particles of pigment-grade TiO_2_ and that oral administration of this pigment can lead to pre-cancerous lesions of the colon, termed aberrant crypt foci, in about a third of WT animals but not in controls without TiO_2_ exposure [37]. In that work, intestinal mucosal levels of TNF-α and IL-1β were modestly increased for animals fed TiO_2_ versus controls [37]. Whilst our data support these findings from a cell culture perspective they also show that particle dose is critical as a determinant of the cytokine response. The precise pathway of TiO_2_ uptake by intestinal cells is still not understood, but it is likely that particles in the lumen have their surfaces ‘decorated’ by soluble molecules of the intestinal lumen so that conjugates (with MAMPs for example), rather than pristine particles, are seen by intestinal cells. Moreover, it is not clear how basal macrophages of the human Peyer’s patches become loaded with particles such as TiO_2_ as, following M cell uptake, particles should be scavenged by phagocytes that are more apical than the observed basal tissue-fixed macrophages [38]. However, despite the pathway not being fully elucidated, the important point is that macrophages of the Peyer’s patches *do* accumulate TiO_2_ particles in humans [9, 39]. If, as we show here, certain genotypes require a lesser cell dose of particles to respond in a pro-inflammatory fashion compared to other genotypes then, in vivo, the initiation of a cascade of inflammation may be host-dependent as well as dose-dependent.

### Specificity of the IL-1β adjuvant effect to TiO_2_ nanoparticles

The ‘role’ of the TiO_2_ particles in the work presented here involves boosting the pro-inflammatory effects of MAMPs via particle-activation of the inflammasome. Many materials activate the inflammasome, including other (nano)particles, and some of these will be more potent than pigment-grade TiO_2_ given the modest efficacy of the latter. For example, MSU crystals and silica particles are activators of the inflammasome (as exemplified here (Fig. 8) and [28, 32, 33]) and have direct relevance in terms of human exposure. MSU crystals may precipitate ectopically and are the cause of joint inflammation in patients with gout, whilst silica exposure to the lungs is well established as an occupational hazard that leads to silicosis in miners. However, in terms of an adjuvant effect on MAMP-primed cells, TiO_2_ deserves particular scrutiny because (a) humans are widely exposed to it orally [3, 5], (b) MAMPs are ubiquitous at high concentrations in the intestinal lumen which is unlike anywhere else in the body, and (c) pigment-grade TiO_2_ is one of two major particle types that accumulates in intestinal (Peyer’s patch) macrophages [7, 9, 39]. The second major particle type, namely aluminosilicate which is mostly in the kaolinite form [7], has not been obviously linked to inflammasome activation although this merits further careful assessment as prolonged macrophage exposure to kaolinite leads to modest IL-1β secretion even in the absence of MAMPS [40].

Interestingly, Winkler et al. have shown that food-grade silica induced production of pro-IL-1β and secretion of mature IL-1β when dendritic cells were exposed to these particles [41]. In other words, silica particles have the capacity to both prime IL-1β formation in the precursor (pro-) form and to induce cleavage to a mature form via inflammasome activation. Although, unlike TiO_2_, this silica has not been demonstrated to accumulate in human intestinal immune cells [7], further studies are merited as there is significant oral exposure and perhaps intestinal cells other than those that have been so far characterised for particle accumulation in the intestine are impacted.

In summary for this section, pigment-grade TiO_2_ is especially relevant as a potential inflammasome adjuvant in intestinal tissue because of human exposure, accumulation, *and* activity. However, other particles, such as aluminosilicates and silica, should not be ignored as there is certainly exposure and accumulation for the former and exposure and potential for activity for the latter.

### IL-1β secretion is not a simple consequence of TiO_2_-induced cell death

Non-biological particles in a size range that enables phagocytosis, which includes pigment-grade TiO_2_, are readily engulfed by macrophages and accumulate in lysosomes [7, 9, 42]. This in turn leads to lysosomal membrane disruption which is a trigger for two concomitant events. The first is cathepsin-dependent IL-1β release which requires inflammasome activation, and the second is cell death which again is cathepsin-dependent but is independent of the inflammasome [42]. Hence, as expected, both events were observed in this study in a dose-dependent fashion when cells were exposed to TiO_2_. In vivo, cell death can lead to pro-IL-1β leakage into the extracellular environment and its activation through ‘alternative’ pathways, such as cathepsin C-neutrophil proteases. However, this does not occur in ‘clean’ cell culture media in vitro [42]. Moreover, a short ‘pulse and chase’ routine protects against such longer term complications. It is therefore anticipated that our observed IL-1β-inducing effect of TiO_2_ in LPS-primed macrophages is independent of the concomitantly observed cell death. Regardless of mechanism, it does not alter the potential relevance of these findings to the in vivo situation where, as noted, pigment-grade TiO_2_ accumulates in selective intestinal cells of humans.

### In vivo relevance for health and disease

Notwithstanding the above, and as discussed earlier, TiO_2_ is only a modest activator of the inflammasome, so whether realistic oral exposure to TiO_2_ leads to interactions with MAMPs and whether intestinal cell loading of both materials is sufficient to trigger inflammation merits closer attention in a relevant genetically susceptible model. In particular, such work should focus on (a) the Peyer’s patches as sites of cellular TiO_2_ accumulation with the potential for early inflammatory processes [11] and (b) the colon, given the association of large bowel cancer with early inflammation and potential exacerbation of disease by TiO_2_ [43].

In addition, our specific interest concerns inflammatory bowel disease, especially Crohn’s disease, and the potential for TiO_2_ as an adjuvant for pro-inflammatory responses in recipient Peyer’s patch cells [7, 38, 39]. Although the murine model used here does not accurately mimic Crohn’s type mutations for *NOD2* because of the duplication of the 3′-end of the WT *Nod2* locus [21], it does, nonetheless, have a heightened susceptibility to inflammation in response to certain MAMPs, precisely as has been proposed for Crohn’s disease [44]. Further work with patient samples is therefore merited to scrutinise the potential for a TiO_2_ adjuvant effect on MAMPs in terms of IL-1β secretion.

## Conclusions

In summary, in this study we have shown that dietary TiO_2_ particles have an impact on the production of the pro-inflammatory cytokines IL-1β and TNF-α by LPS pre-stimulated murine macrophages in vitro, and that TiO_2_ particles can act as IL-1β-inducing adjuvants for bacterial MAMPs that contain MDP moieties. We also demonstrated that the impact of this adjuvant effect is genotype-dependent. Primed macrophages from *Nod2* ^m/m^ mice showed an elevated IL-1β response to incubation with TiO_2_ particles and peptidoglycan compared to cells from WT mice. Further work will need to consider if any human genotypes (sub-populations) are at greater inflammatory risk than the background population from TiO_2_ exposure.

## Additional files

Additional file 1: Flow cytometry gating strategy. (DOCX 321 kb)

Additional file 2: F4/80 expression of LPS-primed BMDMs after chasing with TiO2 +/−peptidoglycan or MDP. (PDF 129 kb)

## Abbreviations

ANOVA: Analysis of variance
BMDM: Bone marrow-derived macrophage
BSA: Bovine serum albumin
FBS: Foetal bovine serum
IL-1β: Interleukin 1β
LPS: Lipopolysaccharide
MAMP: Microbial-associated molecular pattern
MDP: Muramyl dipeptide
MFI: Mean fluorescence intensity
MSU: Monosodium urate
NLRP3: NLR family pyrin domain-containing 3
NOD2: Nucleotide-binding oligomerisation domain-containing 2
*Nod2*^m/m^: Homozygous *Nod2* gene mutation (as described)
PBMC: Peripheral blood mononuclear cell
PBS: Phosphate-buffered saline
PGN: Peptidoglycan
PI: Propidium iodide
pro-IL-1β: pro-interleukin 1β
SD: Standard deviation
SEM: Standard error of the mean
SNP: Silica nanoparticle
SSC: Side scatter
TCM: Tissue culture medium
TiO_2_: Titanium dioxide
TNF: Tumour necrosis factor
WT: Wild-type
