# Supplementary material for: Pro-inflammatory adjuvant properties of pigment-grade titanium dioxide particles are augmented by a genotype that potentiates interleukin 1β processing
Source: Part Fibre Toxicol. 2017 Dec 8;14:51. doi: 10.1186/s12989-017-0232-2 (PMC5721614; doi:10.1186/s12989-017-0232-2)

**Additional file 1. Flow cytometry gating strategy.**

LPS pre-stimulated BMDMs that were exposed to TiO_2_ with or without MDP/PGN were analysed using flow cytometry. At least 12,000 events were acquired per sample. Shown are representative frequency dot plots of WT cells incubated for 3 h in TCM alone (Figure Additional File 1). Each dot indicates a single detected event, i.e. a cell or cell fragment. A colour range from blue to red indicates the amount of dots on the same spot; blue represents a single event, red represents many events. Pink lines indicate analysed areas (so-called gates) with the percentage of events detected in the respective gate shown in the top corners. **(A)** Frequency dot plot of forward scatter against SSC. Shown are only events with a forward scatter intensity of more than 200. These events represent cells and were selected for further analysis. A forward scatter of less than 200 indicates cell fragments and debris. **(B)** Frequency dot plot of PI fluorescence against forward scatter. Cells were classified as PI^−^ when the PI fluorescence was less than 10^1^ on a logarithmic scale. These cells were considered as viable cells and selected for further analysis. Events with a PI fluorescence of more than 10^1^ were considered as dead cells. **(C)** Frequency dot plot of F4/80 fluorescence against forward scatter. F4/80 expression against forward scatter was assessed for all PI^−^ cells. Cells were classified as F4/80^+^ when the F4/80 fluorescence was more than 10^1^. These cells were considered as BMDMs and their SSC intensity was determined.

**Figure Additional file 1**


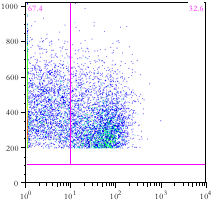


Forward scatter

SSC

PI

Forward scatter

F4/80

Forward scatter

**A**

**B**

**C**


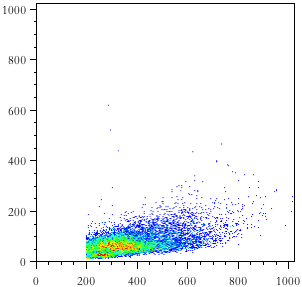

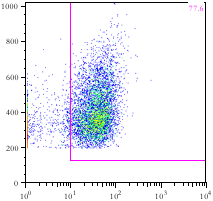

Supplement: Supplementary file 1 — Flow cytometry gating strategy. (DOCX 321 kb) [file 12989_2017_232_MOESM1_ESM.docx]
