# Supplementary material for: Pro-inflammatory adjuvant properties of pigment-grade titanium dioxide particles are augmented by a genotype that potentiates interleukin 1β processing
Source: Part Fibre Toxicol. 2017 Dec 8;14:51. doi: 10.1186/s12989-017-0232-2 (PMC5721614; doi:10.1186/s12989-017-0232-2)
Supplement: Supplementary file 2 — F4/80 expression of LPS-primed BMDMs after chasing with TiO2 +/−peptidoglycan or MDP. (PDF 129 kb) [file 12989_2017_232_MOESM2_ESM.pdf]

Additional file 2. F4/80 expression of LPS-primed BMDMs after chasing with TiO_2 +/− peptidoglycan or MDP.

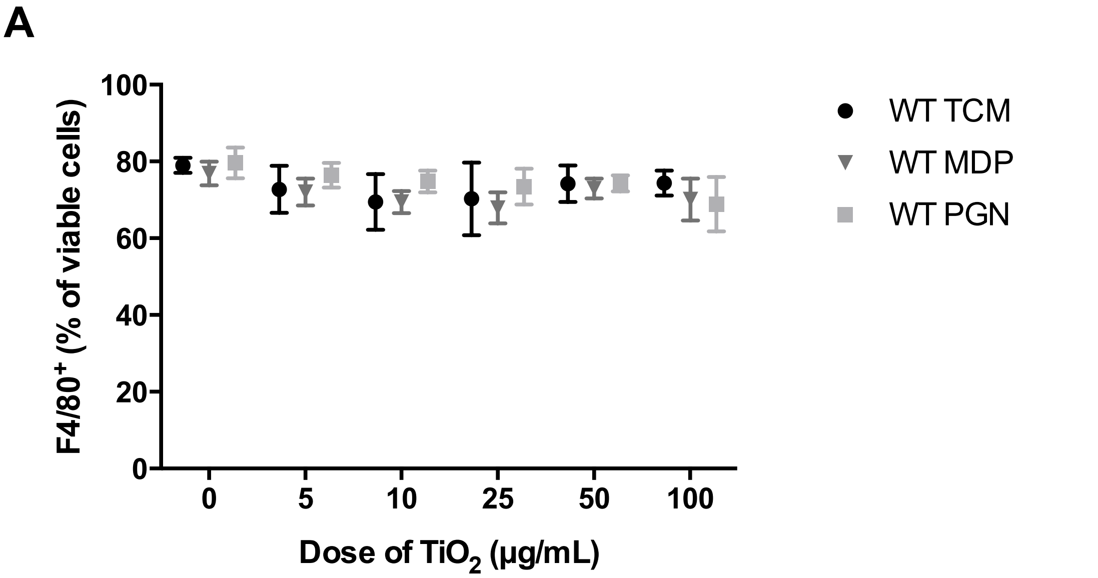

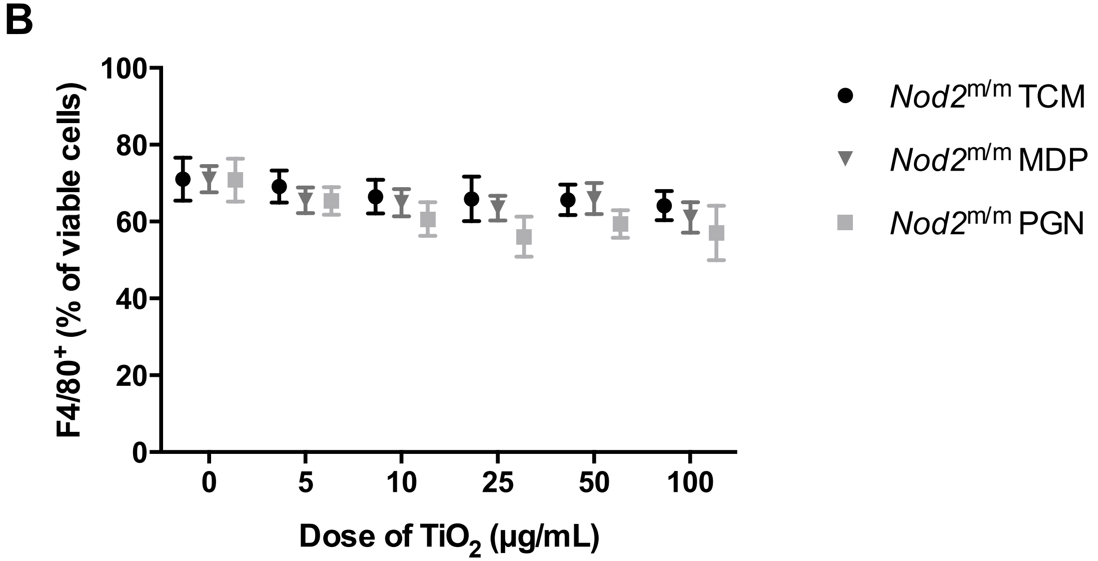

Fig. Additional file 2 BMDMs from WT **(A)** and *Nod2*$^{m/m}$ **(B)** mice were pre-stimulated for 3 h with LPS (10 ng/mL). Then BMDMs were incubated for 3 h with the indicated concentrations of food- and pharmaceutical-grade anatase TiO_2 particles suspended in TCM alone (TCM), TCM + 10 μg/mL MDP (MDP), or TCM + 10 μg/mL peptidoglycan (PGN). Cells were analysed with flow cytometry, and F4/80 expressions of PI^- cells were recorded. Data represent mean ± SD from two independent experiments with three replicates each, *n* = 6.
